# Musculoskeletal pain stakeholder engagement and partnership development: determining patient-centered research priorities

**DOI:** 10.1186/s40900-020-00192-8

**Published:** 2020-06-01

**Authors:** Jason M. Beneciuk, Dorothy Verstandig, Chuck Taylor, Doug Scott, Joan Levin, Raine Osborne, Joel E. Bialosky, Trevor A. Lentz, Tava Buck, Anita L. Davis, Christina Harder, Monika B. Beneciuk, Virgil Wittmer, James Sylvester, Robert Rowe, David McInnes, Tad P. Fisher, Lisa McGarrie

**Affiliations:** 1 Department of Physical Therapy, University of Florida, Gainesville, Florida USA; 2 Brooks Rehabilitation and University of Florida College of Public Health & Health Professions Research Collaboration, Jacksonville, Florida USA; 3 Patient partner, Jacksonville, Florida USA; 4 Brooks Rehabilitation, Jacksonville, Florida USA; 5 Duke Clinical Research Institute and Department of Orthopaedic Surgery, Duke University, Durham, North Carolina USA; 6 Mayo Clinic, Jacksonville, Florida USA; 7 Brooks Rehabilitation Behavioral Medicine, Jacksonville, Florida USA; 8 Brooks Rehabilitation, Jacksonville, Florida USA; 9 Community partner, Jacksonville, Florida USA; 10 Brooks Rehabilitation, Institute of Higher Learning, Jacksonville, Florida USA; 11 St. Vincent’s Family Medicine Residency Program, Ascension St. Vincent’s, Jacksonville, Florida USA; 12 Florida Physical Therapy Association, Tallahassee, Florida USA; 13 Georgia Health Policy Center, Georgia State University, Atlanta, Georgia

**Keywords:** Patient and public involvement, Stakeholder engagement, Communication, Shared decision making, Musculoskeletal pain

## Abstract

**Background:**

Musculoskeletal (MSK) pain is a global public health problem with increased societal burden. Increased attention has focused toward patient and other stakeholder perspectives when determining future MSK pain research priorities, however infrastructure and capacity building within the community are needed for individuals and organizations to participate in patient-centered outcomes research. The purpose of this manuscript is to describe our collaborative experiences with several MSK pain stakeholders and processes to identify a top priority research topic.

**Methods:**

Lunch meetings and formalized workshops were used to develop infrastructure for engaging patients and other stakeholders with early capacity building for partners to identify MSK pain research ideas based on their personal experiences. Additional capacity building and engagement through literature searching further prepared partners to contribute informed decisions about MSK pain research topics and subsequent selection of an important research question.

**Results:**

Several key deliverables (e.g., Governance Document, Communication Plan) were developed and completed over the course of this project to provide partnership structure. Other key deliverables included a list of preliminary comparative effectiveness research ideas (*n* = 8) and selection of shared decision making for MSK pain as the top priority research topic with patient partners identifying pain self-efficacy as an important outcome domain.

**Conclusions:**

Our patient partners provided the catalyst for identifying shared decision making as a high priority research topic based on a wide spectrum of stakeholder perspectives and unique experiences. Patient partners were primarily identified using a single rehabilitation health system and clinician partners were heavily weighted by physical therapists which may have introduced selection bias.

## Plain English summary

Chronic musculoskeletal pain is the leading cause of disability in the United States associated with increased burden for patients, family members, healthcare providers, and society. Musculoskeletal pain experiences are unique to each specific patient, therefore recommendations for increased patient engagement and participation in sharing their personal experiences early during research proposal development has been suggested by other stakeholders. This project focused on developing groundwork and capability for identifying musculoskeletal pain research priorities and specific questions that engaged patient and stakeholders as research partners. The purpose of this manuscript is to describe: 1) our experiences in developing and cultivating a focused partnership consisting of patient and stakeholder partners and 2) processes used to identify relevant research ideas and our top priority research topic. Several key deliverables (e.g., Governance Document, Communication Plan) were developed and completed over the course of this project to provide partnership structure and accountability. Other key deliverables included a list of preliminary research ideas and selection of shared decision making between patients and healthcare providers as the top priority research topic with patient partners identifying efficacy of self-management of pain as an important outcome.

## Background

Musculoskeletal (MSK) pain is a leading global cause of disability associated with increased burden for patients, family members, healthcare providers, and society [1, 2]. For example, in 2015, low back and neck pain was the fourth leading cause of disability worldwide following ischaemic heart disease, cerebrovascular disease, and lower respiratory infection [1]. Between 2005 and 2015, the global prevalence of low back, neck, and other MSK disorders (more than 3 months) has increased 17.3, 21.1, and 20.7%, respectively [2]. In 2016, among 154 conditions, low back and neck pain had the highest amount of health care spending (estimated at $134.5 billion) in the United States, followed by other MSK disorders which had the second highest amount of health care spending (estimated at $129.8 billion) [3].

Current clinical practice guidelines provide recommendations for treating patients with MSK pain conditions primarily based on research findings and clinical expertise, however patient and other stakeholder perspectives are frequently overlooked. MSK pain experiences are unique to each specific patient, therefore recommendations for increased patient engagement early during research proposal development has been suggested [4–6]. Practical recommendations for patient engagement include involvement of at least two patient research partners in all phases of research to ensure the patient perspective is preserved [5], while also acknowledging there is no common methodology that fits every research context [6]. This has potentially important implications since research that is important to patients may provide opportunities to advance patient-centered treatment approaches [7].

In the United States, the prevention and management of chronic pain has emerged as a high priority public health crisis since the release of the 2011 Institute of Medicine report “Relieving Pain in America” [8]. In 2016, the National Pain Strategy provided a broad plan for changing how the nation perceives and manages pain with specific objectives pertinent to several topics including ‘population research’, ‘prevention and care’, ‘disparities’, ‘service delivery and payment’, ‘professional education and training’, and ‘public education and communication’ [9]. In 2018, the Federal Pain Research Strategy provided further specific recommendations by identifying top research priorities in alignment with the National Pain Strategy [10]. Collectively, increased attention has focused toward patient and other stakeholder (i.e., family members, clinicians, insurance companies, health systems, professional organizations, and training institutions) perspectives when determining future research priorities. However, infrastructure and capacity building efforts within the community are required for individuals and organizations to be better able to participate in patient-centered outcomes research [11, 12].

Our partnership was formed and aimed to collectively develop and advance a sustained collaborative effort towards improving management of MSK pain using pragmatic, real-world research methods. We sought to develop an infrastructure for identifying multi-stakeholder pain research priorities and specific questions that engaged a diverse group of partners [13] including patients, community members, researchers, healthcare providers and other relevant MSK pain stakeholders. Early converging themes identified by stakeholders included special interest in non-pharmacological approaches as first-line treatment options and improving communication between patients and providers. Therefore, the purpose of this manuscript is to describe: 1) our experiences in developing and cultivating a focused partnership consisting of several MSK pain stakeholder entities (including patients) and 2) processes used to identify relevant research ideas and our top priority research topic.

### Theoretical background

Collectively, patient engagement in healthcare research that is initiated early during proposal development has been suggested [6, 14, 15]. Lessons learned from patient and stakeholder engagement in pilot projects highlight the importance of continuous and genuine partnerships, strategic selection of stakeholders, and accommodation of stakeholders’ practical needs [16, 17]. Moreover, engaged research partner roles have included research question refinement, selection of interventions to compare, choice of study outcomes and how they are measured, and ensuring studies are patient-centered [18]. For the purpose of this project, we defined patient and stakeholder partner involvement as providing meaningful perspectives and exchanging ideas relevant to improving treatment of individuals with MSK pain conditions (as reflected in our Governance Document).

The framework for patient and stakeholder partner involvement was guided by the Patient-Centered Outcomes Research Institute (PCORI®) Engagement Rubric [19]. Our project specifically focused on research infrastructure and capacity building; therefore, PCORI® Engagement Rubric was used to define potential engagement activities involved during study planning (i.e., developing the research question and relevant outcomes to be studied and defining the characteristics of study participants) [13]. Natafgi and colleagues [4] recently described three levels of early patient engagement (i.e., passive, tokenistic, and authentic engagement) during research proposal development that were influential in describing our experiences. In retrospect, we sought to achieve *‘authentic engagement’* where the patient is a ‘true partner’ in research as evidenced by serving as a co-investigator, joint decision maker, and encouraged to become involved with initiatives leading to patient-driven research [4].

The Guidance for Reporting Involvement of Patients and the Public (GRIPP2) was referenced during manuscript development to assure all relevant criteria were reported when applicable [20]. Specifically, GRIPP2 was beneficial when developing this manuscript by providing clarity for describing the process and context of patient and public involvement (PPI) used during this project.

## Methods

### Patient-Centered Outcomes Research Institute (PCORI®) Pipeline to Proposal (P2P) Program

The PCORI® P2P Program emphasized capacity building and engaging a diverse group of partners including patients, caregivers, community members, researchers, providers and any other relevant stakeholder around a healthcare research interest. Partners were encouraged to develop, propose and implement initiatives that: 1) engage patients as partners, 2) build capacity among all partners to make informed decisions in the research development process, and 3) design research that is focused on patient-centered outcomes. Ultimately, the goal was for the partnership to be well positioned to engage the right partners and collectively determine a comparative effectiveness research question to develop a fundable patient-centered outcomes research proposal. Our project focused on MSK pain and consisted of two separate phases. Tier I (August 1, 2016 to April 30, 2017) focused on developing infrastructure (e.g., Governance Document, Recruitment Strategies Plan) to engage patients (based on their real-world lived experiences) and other stakeholders and early capacity building for partners to identify general themes about MSK pain research ideas. Tier II (August 1, 2017 to July 31, 2018) focused on further capacity building and engagement among stakeholders for providing informed decisions about MSK pain research topics through literature searching and subsequent selection of an important and meaningful comparative clinical effectiveness research question.

### Data collection

For Tiers I and II described in greater detail below, we did not incorporate a systematic process for data collection. Rather, we had our research manager transcribe key dialogue that was presented during lunch meetings and formal workshops. For example, patient testimonials described below were transcribed as direct quotes and results of any voting were documented accordingly. Over the course of the project (and as per recommendations from our Technical Assistance Office (TAO)), we attempted to delegate this ‘note taking’ responsibility to other partnership members as a strategy to enhance engagement.

### Partnership development – Tier I

Early partnership development focused on identifying clinician and patient stakeholders with invested interest and willingness to provide their perspective about past MSK pain related healthcare experiences. For convenience, we initially utilized a single health system (Brooks Rehabilitation, Jacksonville, Florida) to engage clinicians and patients as partners of our team (i.e., not research subjects). Briefly, Brooks Rehabilitation (BrooksRehab.org) is a post-acute rehabilitation health system providing a System of Care approach to advancing the health and well-being of individuals in their communities. Rehabilitation services are delivered in a variety of settings including inpatient rehabilitation, skilled nursing, assisted living and memory care, home health, community programs, outpatient rehabilitation, and a physician practice. We initially targeted physical therapists from outpatient rehabilitation settings that routinely provided care for patients with MSK pain conditions. These physical therapists were then encouraged to engage current or former patients that may also be interested in participating. New patient partners were then encouraged to disseminate project efforts in their communities in an attempt to broaden the partnership (i.e., snowball sampling) [21]. Later development consisted of identifying and engaging with other stakeholder entities not yet represented in the partnership (i.e., professional organizations, family medicine, private insurance companies) with recruitment strategies primarily consisting of in-person meetings.

### Partnership characteristics

Our partnership consists of a diverse collection of patient partners and other MSK pain stakeholders in an effort to ensure a variety of perspectives. We purposely attempted to develop a partnership that was similarly distributed in the number of patients, clinicians, and researchers to ensure an equal number of different stakeholder entities.

#### Patient partners

Our patient partners (*n* = 6) were individuals with consistent dissatisfaction for current or past lived pain related healthcare experiences and had strong opinions for improvement. Patient partners were primarily identified through purposive sampling [21] and were targeted based on poor healthcare experiences as we believed this approach provided the greatest opportunity to identify areas for improvement [4]. However, we do acknowledge that this may have introduced selection bias and their perspectives may not be generalizable to all patient experiences [22]. Patient partners were identified by direct communication with a physical therapist although not all were receiving current care. Patient partners had sought healthcare for a variety of pain related conditions in several clinical settings including primary care, family medicine, rheumatology, physical therapy, chiropractic, and orthopedic surgery. Several patient partner testimonials are provided in Appendix.

#### Clinician partners

Our clinician partners (n = 6) were treatment providers from a variety of healthcare disciplines (physical therapy, clinical psychology, family medicine) that routinely provide care for patients impacted by MSK pain conditions. Most clinician partners were affiliated with a single rehabilitation health system (Brooks Rehabilitation, Jacksonville, Florida), however our physician partner was affiliated with a separate large health system located in Jacksonville, Florida. Physical therapists provided care in outpatient (*n* = 3) or pain rehabilitation (*n* = 1) settings and our clinical psychologist (*n* = 1) specialized in pain rehabilitation. Our physician partner served as program director for a family medicine residency program.

#### Research partners

Our research partners (*n* = 4) were individuals affiliated with a formal research collaboration between Brooks Rehabilitation (Jacksonville, Florida) and the College of Public Health and Health Professions at the University of Florida (Gainesville, Florida). Research stakeholders were selected based on having special interest in MSK pain with expertise in the areas of clinical decision making, health services research, and education.

#### Other stakeholder partners

Other stakeholder entities were individuals from a state level healthcare professional organization (n = 1), community health advocacy collaborative (n = 1), rehabilitation education institute (n = 1), family medicine residency program (n = 1), and national private health insurance provider (n = 1) to provide diverse perspectives and further contribute to potential research areas of focus and specific question development.

#### Special partnership roles

An Advisory Board Group was formed with equal distribution across patient (*n* = 3), clinician (*n* = 2), and research (n = 2) partners. These individuals were identified as partnership leaders (expressed desire to take on leadership roles) and expected to actively contribute to project efforts by: voting on key decisions (while also considering other group member perspectives), communicating with other potential stakeholder collaborators, and contributing intellectual perspectives during meetings and workshops. Criteria were established to increase Advisory Board Group accountability (Table 1). An Active Partner Group was also formed to provide stakeholders with opportunities to provide perspectives and contribute in partnership activities (e.g., lunch meetings, workshops) despite not being able to commit to specific roles and responsibilities designated for Advisory Board Group members. Our intention was to provide opportunities for all group members to participate in activities and be involved with key decisions, however Advisory Board Group members were ultimately involved with voting on key decisions.

**Table 1:** Special Partnership Roles and Accountability Criteria

	Roles	Accountability
Advisory Board Group	• Voting on key Partnership decisions • Communicate with other potential stakeholder collaborators • Contribute intellectual perspective during meetings and discussions	• Commitment to 12-month term • Commitment to attend ≥75% of group meetings (in-person or remotely) • Communicate with ≥2 other potential stakeholders in effort to establish new collaborative opportunities • Represent Partnership during community group presentations
Active Partners Group	• Non-voting role • Contribute intellectual perspective during meetings and discussions	• Commitment to 12-month term • Commitment to attend ≥50% of group meetings (in-person or remotely)

### Partnership engagement methods – Tier I

The primary goals for Tier I were to develop infrastructure for engaging patients and other stakeholders and early capacity building for partners to identify general themes about MSK pain research ideas based on their real-world lived experiences. We primarily used two platforms to engage partners and share information (described below) with several key deliverables (Table 2) expected prior to advancing to Tier II. We intentionally made efforts to assure equal voice across different stakeholder groups were provided by promoting a comfortable and respectful environment during group interactions.

**Table 2:** Key Project Deliverables

	Deliverable	Description
Tier I	Recruitment Strategies Plan	Preliminary agreement among partnership about plans to broaden the community of patients and other stakeholders who may be interested in participating in or supporting project efforts. This document also clarified types of individuals and organizations we needed to recruit that would optimize project success while also meeting expectations of our funding agency. Obtaining diverse perspectives from patient, clinician, researcher, and other stakeholders was vitally important.
	Governance Document	To describe partnership infrastructure and optimize stakeholder engagement during project. This document consisted of several components including: 1) vision, mission, and purpose statements; 2) membership requirements; 3) membership expectations; 4) decision making processes; 5) operational guidelines; and 6) communication between partners. Several drafts were circulated until consensus agreement^a^ was achieved with expectations for future modification as needed. Operational accountability statement: “*Each member, from researcher to patient, is accountable to the Partnership, to each other and to the vision, mission and purpose of the Partnership. We each have a responsibility to suit up, show up and speak up*.”
	CER Ideas	Focused list of comparative effectiveness research ideas relevant to musculoskeletal pain and reflective of all stakeholder perspectives was developed. Partners were asked to brainstorm about potential ideas prior to future formal meetings and subsequent literature review activity.
	Formal Advisory Board	Consisted of a diverse mix of patients (*n* = 3), clinicians (*n* = 2), and researchers (*n* = 2) that were identified by project lead (JMB) as those who were ideal for providing valuable contributions based on early team interactions. Advisory Board member roles included: voting on key partnership decisions, communicating with other potential collaborators, and continued contribution of intellectual perspectives during formal meetings and discussions.
Tier II	Communication Plan	To mature and formalize partnerships developed through the Recruitment Strategies Plan (Tier I). This document described how partnership will communicate internally and externally, who will be responsible for activities, how often will communication occur, and what messages and modes of communication will be used. Expectations were this document would be modified over time.
	CER Question	To develop a formal CER question, the PICOTS framework was used to describe the study population, intervention, comparator, outcomes, timeframe, and setting. Patient, clinician, and research stakeholders played an active role in this process.
	Growth & Sustainability Plan	The intention of this document was to serve as a guide for our partnership to discuss and complete its plan to communicate with and use resources from other stakeholders beyond P2P funding with description about how these partners would help support future efforts (e.g., grant proposals).
	Proposal Opportunity Plan	Served to track potential funding opportunities, pending applications, etc.

*CER* comparative effectiveness research. ^a^ indicates consensus decision-making process used for Advisory Board Group members to identify and to come to majority agreement by identifying and exploring all member interests. All Advisory Group members (*n* = 7) will be required to participate in order to achieve a simple majority consensus agreement of at least 4/7 votes. However, we realize that this may be problematic in the event where an Advisory Board Group member may not be able to vote based on individual circumstances or in the event of a vacant position. In these cases, simple majority voting will be based on greater or equal to 51%. The partnership will operate with the recognition that the needs of all stakeholders affected by a decision need to be included in the deliberations

#### Lunch meetings

Monthly lunch meetings were scheduled to enhance partnership engagement opportunities and maintain consistent face-to-face communication between team members [17]. Lunch was provided for all participants that attended with remote options made available for those not able to attend in-person. These meetings also provided an administrative platform for continued conversation about previous interactions (e.g., workshop topics) with the overall intent being to enhance stakeholder engagement, sustain project motivation, and assure compliance to project timelines. Specifically, stakeholder decision making for determining partnership structure and direction frequently occurred during lunch meetings through friendly deliberation and consensus voting when necessary.

#### Formal workshops

Between November, 2016 and April, 2017 three formal workshops (each 2 h in duration) were provided each serving distinct purposes described in detail below. Dinner was provided to all attendees and each participant received a $50.00 gift card.

##### Overview & key Deliverables

The first workshop revisited project efforts discussed in previous lunch meetings, described and clarified individual stakeholder roles (e.g., patient, clinician, and researcher) [23], introduced and distinguished between patient-centered outcomes research and comparative effectiveness research [24] and allowed for discussion about Tier I deliverables (Table 2).

##### Partnership Infrastructure & Direction

The second workshop focused on mid-project report feedback provided from the funding agency. For example, continued documentation of conversations and collective group decision making was strongly encouraged. Time was also dedicated to discussing initial components of the Recruitment Strategies Plan and Governance Document (Table 1). A formal partnership name (*North Florida Musculoskeletal Pain Research Partners*) was determined through deliberation and consensus voting as team members indicated the need for increased visibility and a sense of ownership. In addition, the National Pain Strategy [9] was suggested as a framework for concept building and guiding future partnership decisions. Partnership members were encouraged to review the National Pain Strategy for determining: 1) appropriateness as a guiding resource and 2) relevant objectives that should be considered as partnership focus (i.e., ‘population research’, ‘prevention and care’, ‘disparities’, ‘service delivery and payment’, ‘professional education and training’, and ‘public education and communication’) [9].

##### Stakeholder perspectives

The third workshop allowed individual members to provide unique perspectives about National Pain Strategy objectives. Through deliberation and consensus voting, everyone believed that although ‘*professional education and training*’ and ‘*public education and communication*’ objectives were consistent with partnership interests, focusing on ‘*prevention and care*’ provided greatest opportunities to positively impact clinical practice. To determine if our specific interest in non-pharmacological treatment options for MSK pain were consistent with other stakeholders, we sought perspectives from a state level professional organization (i.e., Florida Physical Therapy Association) guest speaker to provide updates about public policy discussions surrounding prevention and care of opioid use in the state of Florida. A focused list of comparative effectiveness research ideas relevant to MSK pain and reflective of all stakeholder perspectives was developed (Table 3). Partners were asked to brainstorm about potential ideas prior to future formal meetings and subsequent literature review activity.

**Table 3:** Preliminary Comparative Effectiveness Research Ideas

Comparative Effectiveness Research Idea	Stakeholder Presenting Idea
**Secondary prevention of musculoskeletal pain:** Comparing strategies to identify and provide targeted intervention for patients with new episodes of musculoskeletal pain.	Clinicians Researchers
**Tertiary prevention of musculoskeletal pain:** Comparing strategies to identify and provide targeted intervention for patients with chronic pain.	Patients Clinicians Researchers
**Communication Enhancement following Discharge for Musculoskeletal Pain:** Comparing strategies to introduce or enhance communication with patients and family members following discharge from healthcare services.	Patients
**Shared Decision Making for Musculoskeletal Pain:** Compare strategies for shared decision-making (or SDM compared to strategies that do not incorporate SDM).	Patients Researchers
**Healthcare Provider Education:** Compare the impact of informing different healthcare stakeholders on the beneficial role of physical therapy for musculoskeletal pain.	Patients
**Telehealth for Musculoskeletal Pain:** Compare Telehealth strategies (or Telehealth compared to other remote strategies) for musculoskeletal pain management.	Clinicians Researchers
**Physical Therapy Direct Access Education:** Comparing education intervention strategies to inform the public about direct access opportunities for receiving physical therapy for musculoskeletal pain.	Clinicians

### Partnership engagement methods – Tier II

Formal application for Tier II progression was required. The primary goals for Tier II were focused on further capacity building and authentic engagement of patient and clinician stakeholders [4, 14]. Partnership decisions were guided by providing literature search informed decisions about MSK pain research topics and subsequent selection of an important and meaningful comparative clinical effectiveness research question (described in greater detail below). Similar to Tier I, we primarily used two platforms for partnership engagement with several key deliverables (Table 2) expected upon Tier II completion.

#### Lunch meetings

Monthly lunch meetings were again provided to enhance partnership engagement opportunities and maintain consistent face-to-face communication between team members with remote options being provided for those not able to attend in person. Necessity for recruiting additional stakeholders and clarifying specific research question components were main areas emphasized during Tier II lunch meetings.

#### Formal workshops

Between September, 2017 and May, 2018 five formal workshops (each 2 h in duration) were provided each serving distinct purposes described in detail below.

##### Literature searching

The first workshop focused on Tier II application feedback, revisited National Pain Strategy objectives as top priorities, reviewed preliminary comparative effectiveness research (CER) research ideas (developed during Tier I) (Table 3), and introduced literature searching methods. Literature searching was important for providing brief overview about pre-determined research ideas and support for identifying partnership top research priorities, in addition to actively engaging partnership members in the research development process. Results from team literature reviews provided additional insight as to current literature gaps and a method for generating subsequent discussion. Time was dedicated for partners to become familiar with literature searching methods using a variety of search engines. Patient partners were paired with clinician or research stakeholders that had previous literature searching experience and common interest in a MSK pain treatment approach. Expectations were that individual dyad members would communicate outside of workshop to complete literature searching activities. To provide structure, we asked to specifically: 1) identify any previously published systematic reviews; 2) identify at least 5 to 10 peer-review publications published in past 10 years, and 3) provide collective summary of findings to identify potential literature gaps.

##### Top research priority

The second workshop was dedicated to disseminating literature review findings amongst partnership members. After working in teams, patient-clinician (or patient-researcher) dyads shared literature review findings with the larger team to narrow research priorities. Following deliberation, partnership members voted on a single topic with the most apparent gaps in the literature that would serve as top research priority for the remainder of this project.

##### Research question development

The third workshop focused on research question development by introducing the PICOTS framework to describe the study population, intervention, comparator, outcomes, timeframe, and setting [25]. Partnership members began to discuss different PICOT components with further development discussed during subsequent lunch meetings.

##### Patient-centered outcomes

The fourth workshop focused on introducing the concept of different outcome domains and prioritizing which were most important to our patient partners [14]. We focused on self-report outcome measures as our patient partners indicated they have greatest potential for reflecting individual patient perspectives. Research and clinician partners provided examples of and perspectives about different outcome domains (e.g., pain and function) to assure all relevant MSK pain domains that are routinely included in research proposals and clinical practice were considered (e.g., additional healthcare utilization).

##### Letter of intent development

The final workshop was dedicated to drafting a Letter of Intent for an upcoming funding opportunity. Patient, clinician, and research stakeholders all provided unique perspectives during refinement of specific sections including: study title, background, aims, and methods.

## Results

Several key deliverables were developed and completed over the course of this project to provide partnership structure (Table 2). These include, however were not limited to, our Governance Document, Communication Plan, and Proposal Opportunity Plan. Other key deliverables included a list of preliminary comparative effectiveness research ideas (Tier I) and selection of a top research priority topic (Tier II), described in greater detail below.

### Partnership member retention

Over the course of Tiers I and II, all partnership members contributed on some level to ongoing initiatives and activities (i.e., lunch meetings and formal workshops) with the exception of two patient partners and one clinician. One patient partner relocated out of the region and declined remote attendance options, while the other was non-responsive to attempts at follow-up communication. One clinician was not able to participate due to other responsibilities.

### Comparative effectiveness research ideas

MSK pain was identified by the project lead (JMB) as the primary topic of interest, however specific emphasis on the role of non-pharmacological treatment options emerged as high priority during early Tier I stages of partnership development. Seven CER ideas were initially selected (Table 3). Surprisingly, there was a strong degree of alignment focusing on frustrating (and at times alarming) patient or family member healthcare experiences. Patients introduced communication and education focused needs including: 1) enhanced communication following discharge from healthcare providers and 2) coordinated communication among healthcare providers of different disciplines (e.g., medicine, physical therapy) and between providers and patients. Patients acknowledged that although some patients may be more impacted by these shortcomings (e.g., those with less social support and/or low health literacy), they also firmly believed “*these ideas are relevant for all people with pain*”. Clinicians contributed to patient ideas and emphasized the need to better inform the public about physical therapy direct access opportunities. Clinicians (with strong support from researcher stakeholders) also suggested the need to distinguish between secondary and tertiary prevention for MSK pain with special emphasis placed on the role of treatment approaches guided by responses to risk stratification tools [26–29]. As the opportunity for improving communication between patients and healthcare providers emerged as the highest priority topic, research stakeholders suggested the concept of shared decision making as a potential additional topic. Therefore, literature reviews were performed by partnership members for eight total CER ideas (Table 3).

### Top priority research topic

Non-exhaustive literature review findings identified research gaps and following brief deliberation, shared decision making was selected as the top priority MSK pain research topic during Tier II with greatest potential for future impact [30, 31]. Several stakeholders commented that having a structured tool would be valuable for guiding shared decision making discussions between providers and patients. Patient partners also provided strong beliefs about self-efficacy for self-management of symptoms to be considered as an important clinical outcome. Additional relevant outcome domains included patient reported pain and function, high impact chronic pain incidence, additional healthcare utilization, and use of opioid medications (Table 4).

**Table 4:** Patient partner selected outcome domains of interest

Primary outcome	Patient reported outcomes for pain and function
Secondary outcomes	Presence of chronic pain (Yes or No) If yes – severity of chronic pain (Mild, Moderate, Severe) If yes – presence of high-impact chronic pain (Yes or No)
	Self-efficacy for self-management of symptoms
	Additional healthcare utilization
	Use of opioid medication

## Discussion

The overall purpose of this project was to cultivate a sustainable partnership consisting of patients, clinicians, researchers, and other MSK pain stakeholders. Ultimately, our future goal is to be well positioned for developing a high-quality research proposal with a strong engagement plan that leads to externally funded research (short term goal) with strong potential for improving patient-centered MSK pain outcomes (long term goal). Our experience has provided a structured description of how to successfully engage patients and other stakeholders during pre-planning (i.e., development) phases of the research process [4], which may be useful in guiding growth and maturation of other collaborative partnerships.

### Facilitators of partnership development and sustainability

There were also several favorable aspects to this project that improved our ability to develop, cultivate, and sustain our partnership and external collaborations. First, we were very fortunate to have engaged passionate stakeholders for improving how MSK pain is managed in healthcare settings. Moreover, patient and clinician partners were fairly balanced in number with each actively participating in-group discussions by sharing unique perspectives through individual testimonials which were extremely impactful [32]. Second, we established a positive culture of respect and open mindedness during early stages of this project where all stakeholders were willing to listen and learn from one another, which provided a comfortable environment for active partnership dialogue [16, 32]. It was important our patient partners were not intimidated and were respected when sharing their unique perspectives amongst a group of clinician and research stakeholders. An example is best described through a patient partner testimonial: “*during meetings we solicited input from all stakeholders, we wanted to hear their experiences, opinions from patients and reactions from clinicians, there was no rolling of eyes … this was uncomfortable at times to listen, yet important and tremendous value to all stakeholders … clinicians may have never experienced that form of communication with patients.*” On several occasions, deliberation and consensus voting was necessary to resolve conflicting points-of-view surrounding specific topics (e.g., operational definition of shared decision making), however this process ensured all participant perspectives were considered prior to arriving at final decisions. Third, we were able to identify patient partners as leaders of activities within our core Advisory Board Group and ensured they were impactful in these roles. For example, several patients played significant voluntary roles during outreach efforts to other community stakeholders in collaboration with the Florida Physical Therapy Association which exemplified the value of patient voice [33]. Specifically, patient partners provided individual video based perspectives about the value of non-pharmacological treatment options that were made available to the public. The project lead intentionally and routinely communicated with the Advisory Board Group as to their level of comfort in their respective roles; however, we did not provide additional training for this leadership role which is a potential limitation to our approach. Finally, we had external financial and technical support provided by the project funding agency. We were fortunate to be able to compensate patients and other stakeholders for their time dedicated to partnership activities (e.g., workshops, external literature review activities). PCORI® also provided project support for all P2P awards through regional Technical Assistance Offices (TAO). The Georgia Health Policy Center provided support to this award through regular contact with the project (JMB) and patient (DV) lead. Technical assistance focused on supporting the collaborative development of the governance document, completion of deliverables, brainstorming effective engagement strategies, as well as building research team capacity to more authentically and sustainably engage the community in research. Additional TAO activities included providing lessons learned from other regional P2P cohorts and opportunities for disseminating outcomes of this project.

### Barriers to partnership development and sustainability

There were several challenges to developing, cultivating, and sustaining our partnership and external collaborations. First, individual engagement through participation in partnership activities varied between individuals; therefore it was important to focus on strategically developing relationships with high potential for sustainability [17]. Our initial recruitment approach focused on partnership ‘quantity’ as opposed to ‘quality’, which was eventually reconsidered following unsuccessful attempts at achieving buy-in from potential collaborators and recommendations from our TAO. Second, some stakeholder entities are more difficult to engage compared to others [34]. Our partnership specifically struggled in developing collaborative relationships with private insurance companies primarily because we failed to initially identify and engage influential individuals within those organizations. We suggest other partnerships remain persistent and are strategic in developing these important relationships [17], as the influence of insurance company policies and perspectives are vitally important when considering issues that involve potential changes in clinical practice [35–38]. Third, scheduling and communication between meetings was difficult. Providing remote communication options was a strategy used to address this barrier [39], however became challenging as partnership size increased and became more diverse. As partnership demographics continue to diversify, providing remote options alone will not address those without access to technology and other platforms need to be considered. Finally, partnership sustainability after identifying our top priority research topic (shared decision making) and following project funding has been a challenge as we discuss opportunities for securing future funding for a productive research project.

### Shared decision making as a top priority research topic

Our partnership initially had broad interest in promoting non-pharmacological treatment options for MSK pain (particularly because many are associated with low risk of harm) [40–42] and improving communication between patients and treatment providers (specifically because integrating patient preferences for treatment are consistent with patient-centered care) [43, 44]. Shared decision making is a collaborative strategy between patients, families, and providers where healthcare decisions are made using truthful information about treatment options (including benefits and harms) in easy to understand formats to enhance patient engagement opportunities [45, 46]. Therefore, it was not surprising that partnership members were in favor of implementing a standard tool (or model) to enhance shared decision making processes [47]. Although emphasized in recent healthcare initiatives [48], SDM is not commonly integrated into clinical practice with a specific evidence gap in the value added for MSK pain [30].

### Limitations

There are several limitations in regard to methods used for this project that need to be acknowledged. Our patient partners were primarily identified and recruited to participate through a single rehabilitation health system, which may have influenced the decision to focus on shared decision making. Selection bias may have also been introduced as many of our patient partners emphasized poor healthcare experiences and were identified by physical therapists, therefore generalizability to other patients may be limited [22]. Moreover, our patient partners did not represent an extensively diverse demographic profile (e.g., there was no representation from the African American or Latino communities) and our clinician partners were heavily weighted by physical therapists which may have potentially limited opportunities for discussions about other complementary, alternative, or integrative health treatment options (e.g., yoga) [49]. We did not strictly monitor or enforce stakeholders for expected levels of accountability identified in our Governance Document and related to project related activities (e.g., communicating with other potential collaborators). Therefore, we suggest future partnerships establish, monitor, and adhere to succinct expectations so that stakeholders wishing to become more actively involved are held accountable to increase potential for project success. Finally, although our patient partners indicated they believed their individual perspectives were being heard (through direct communication with project lead), we did not include independent facilitators to validate this assumption.

### Future directions

Since identifying shared decision making as our top priority research topic, we have identified an international content expert to provide further guidance in preparation for future grant proposal development. We have continued to cultivate a collaborative relationship with a private insurance company, other local health systems, and strategically identified potential external funding opportunities. We also plan to expand our stakeholder team to include other patient partners from the community, which will allow for demographics that are more diverse and potentially include positive health care experiences.

## Conclusions

Our patient partners’ suboptimal healthcare experiences have provided the catalyst for identifying high priority MSK pain research topics. Our partnership initially had very broad interest in promoting non-pharmacological treatment options for MSK pain and improving communication between patients and treatment providers. Collectively, our capacity building and community engagement efforts have culminated in identifying shared decision making as a high priority MSK pain research topic that was based on a wide spectrum of stakeholder perspectives and unique experiences.

### Supplementary information

**Additional file 1.**

## Data Availability

Not applicable.

## Appendix

### Patient and Community Partner testimonials

#### Please provide your perspective about experiences with our group over the past 2 years

*For the past two years I have participated in this project that requires collaboration between patients, physical therapists, and community stakeholders. Whether discussing research findings, governance issues, possible research questions, the group has benefitted from the varied backgrounds of its members. I feel privileged to have been part of this project.* (Patient Partner 1).

*As our group researched and discussed whether our research question would be narrowed to Shared Decision Making, I learned so much. I became a more assertive patient (although I’ve never been a shrinking violet). I am convinced that, Shared Decision Making is essential, especially for patients dealing with numerous providers, both of whom may not know how to communicate and/or aren’t in the habit of communicating with each other.* (Patient Partner 1).

*Over the past 2 years, I have participated in monthly lunch meetings and quarterly workshops for North Florida Musculoskeletal Pain Research Partners. I am a patient stakeholder and member of the Advisory Board and have been part of the group with a voice and vote from the beginning including the development of the group’s name and focus. I participated in the development of the Governance document including defining the group’s vision, mission, purpose, roles, the weight of the different stakeholders and operational accountability. I have researched evidence for some of the comparative effectiveness research (CER) ideas our partnership has developed. As a patient stakeholder I am able to offer my perspective on the issues we discuss and share what is important to me as a patient with chronic pain. I carry not only my voice, but the voice of other chronic pain sufferers.* (Patient Partner 2).

*Thanks for the article. It was......interesting. What I found interesting was the question itself. The authors basically wanted to know if the internet was providing accurate information. I think the better question is, why are patients going to the internet to get their healthcare questions answered? I think there are at least two primary answers to that. Number one is lack of effective communication between doctors and their patients (and there can be many reasons for that...). Number two is that patients don’t have a lot of faith or trust in their doctors. Can shared decision making change that? If all parties are willing to do their part!* (Patient Partner 2).

*Over the past 2 years I have seen this group flourish and grow beyond what I would have imagined. I was asked to be part of the group as a patient partner not knowing what to expect or how I could contribute. Without having any knowledge of the medical field, I was hesitant to obligate myself to something that I was unfamiliar with or felt unprepared to contribute on any level. From the very beginning, I was welcomed and encouraged to share my thoughts and opinions based on my “real world” experiences. Because this group is patient centered, it is my core belief that this group would not have thrived as well as it has. The clinicians that are part of this group take my thoughts and suggestions seriously and apply them to the objectives of the group. The communication and thoughts discussed at the meetings have proven to be a true team experience with all those involved. During this project, I was invited to attend a professional conference. Since I have no medical background, I found the experience enlightening on many levels. I found different research groups to be dedicated people who passionately believe in their cause as well as interesting and necessary research topics. After returning from the conference, I found myself to be even more dedicated to this group. Having experienced the medical issues I have and other related setbacks due to those medical issues, I have found that this group has given me the opportunity to help others and find ways to better our healthcare systems and treatment options.* (Patient Partner 3).

*I would like to convey my observations as participant in the planning process to prepare a competitive grant application. My perspective is that of a laboratory scientist in the molecular genetics arena and as an interim-CEO of a fledgling health collaborative in the community.* (Community Partner).

*As project lead, you conducted meetings that educated the diverse group in such a way that everyone’s input would be constructive, worthwhile, and considered. The multiple viewpoints were synergistic allowing consensus to develop as time when on. In sum, the step wise process promoted group understanding, commitment, and output continued to grow. You were patient, the participants were faithful and the path enjoyable.* (Community Partner)

#### Based on previous healthcare experiences, please describe your frustration in regard to management of pain

*Over the years it has often been frustrating as a musculoskeletal patient, being treated by a neurologist, rheumatologist, pain management doctor, and physical therapist for related issues often at the same time. However, my providers were not communicating with each other. This meant I had to be the link between them. Shared Decision Making still isn’t a concept most of them understand. They are getting better at it.* (Patient Partner 1).

*After several years of chronic pain and recent pain in my left shoulder, my primary care doctor finally agreed to an x-ray which was normal. I was also seeing a rheumatologist and told her about the pain and she ordered an MRI which showed a frayed rotator cuff. I went to an orthopedic doctor who ordered physical therapy. If my primary had ordered the MRI I would have been treated a lot sooner. I also recently injured my right knee, saw my primary care doctor who ordered an x-ray. It was normal, but I was still having pain. I convinced him to order an MRI, which he did and then referred me to an orthopedic. I then saw the orthopedic and was diagnosed with a torn meniscus and bruised bone in the right knee. He ordered physical therapy which I started more than two months after the original injury. Soon after, I skipped my primary care doctor and went straight to the orthopedic for decreased range of motion in my neck and pain in my mid-back. More physical therapy. Then, just as things were getting better, a very large man stepped back into and on me. I reinjured my right knee as well as injuring my left wrist and right ankle and foot. I went back to my orthopedic, thinking he could see me for all three injuries. Not so. Three different areas meant three different doctors. More physical therapy. I am currently struggling with pain in my mid-back. As usual, the x-ray is normal. When I received the report, I should have been told what the next diagnostic step is, because the pain is still present. Instead, I will have to call and request an order for an MRI. The delay in treatment is not only frustrating, it’s time consuming and exacerbates the original injury. For me, that means it exacerbates other comorbidities as well. Another frustration is I have to be cognizant of how many physical therapy visits I have used, so I don’t run out before the end of the year and my insurance stops paying.* (Patient Partner 2).

*Treatment has almost always been delayed because clinicians have not been proactive (a normal x-ray doesn’t mean nothing is wrong) and it has taken multiple referrals to see the appropriate doctor … The biggest barrier I have found is the fact that most insurance companies do not pay for anything other than conventional medicine, and even that is limited. They will pay for medication forever, but you can only have so many PT visits. FYI, I am not taking any medications, prescription or OTC (*i.e.*, over the counter) (partially personal choice and partially because they cause other problems).* (Patient Partner 2).

*The management of musculoskeletal pain in my opinion has not been properly addressed by a majority of the medical field. My experience with musculoskeletal pain was due to 2 car accidents. I was instructed to see a chiropractor for my low back and neck pain. During those visits, the chiropractor had a visiting pain management physician in his office. The pain management physician’s course of treatment was to prescribe pain medication which started with muscle relaxers and eventually progressed to prescribing opioid medication, Oxycodone and Morphine. I was told by the pain management physician that I should accept the fact that I will be taking those medications possibly for the rest of my life. Had I accepted his prognosis, I would not be alive today. Instead, I was lucky to have a friend who knew the director of the chronic pain program at Brooks Rehabilitation and they were able to remove my dependence from the opioid medication and showed me that physical therapy will help me recover from my injuries. My story is not unique. It seems to me that medical professionals use opioid medication as a way to temporarily relive the symptom and appease the patient.* (Patient Partner 3)

#### Based on your previous experiences, why do you believe it is important to be part of this group?

*My importance to the group was the perspective I shared as a patient with musculoskeletal issues.* (Patient Partner 1).

*It’s important to be a part of this group because I believe the patient’s perspective is as important, if not more so, than any of the other stakeholder’s. This particular group allows me to have a voice and a vote in decisions that may ultimately directly affect my healthcare and other’s like me. I believe our healthcare system needs to change so that the patient is the primary focus, not the insurance companies and the policy makers. With all of my issues, I believe I have a unique perspective that is important for the other stakeholders to hear and see. I am an example of what chronic pain does; difficulty with movement, memory, focus, patience, activities of daily living.* (Patient Partner 2).

*This is important to me because I am literally in pain … ..all the time. In theory, shared decision making would probably be of great benefit to the patient so yes, I think we are heading in the right direction. However, in reality, shared decision making is going to take too much time that doctors don’t have. Most doctors just want you to do what they tell you without question. Also, unless it’s going to produce money for the insurance companies, the regulatory agencies, the doctors, and every other organization involved … nothing is going to change. Is that a cynical attitude? Yes it is, but in my experience, healthcare is no longer about providing what is needed for the patient. It’s about providing power and financial gain for the policy makers.* (Patient Partner 2).

*Because this group is patient centered, I believe it’s important to use my experience with opioid medications and physical therapy to better understand the need for alternative treatment plans. I want the hardship and troubles I experienced to be beneficial to the group and the research this group performs. I feel that I have beat the odds regarding the consumption of opioid medication and I want to use that experience in a positive way to change how musculoskeletal pain is diagnosed and treated. The opioid epidemic has had an unprecedented catastrophic effect on our society and I believe my experience with this group can help change that.* (Patient Partner 3).

*Why was it important to part of this group? As this phase is completed I now reflect on the value of my participation. It is my belief that research advances are more robust when multiple perspectives, experiences, and expertise to merge to address a problem especially in clinical fields where evidence-based practices should be adopted. Secondly, when a clinical sector is pursuing or testing a new direction, there is much to be learned from their issues that could be enhanced or generalized. Therefore, local health advancements require some level of cross contamination, cross participation such that the whole is greater than the sum of its parts. I can only hope that my past, present and future input helped move the needle, not only for North Florida Muscular Skeletal Pain Research directly but also for other health sectors with whom I will intersect.* (Community Partner)

#### Please describe what you would like to see result from our group efforts over the past two years (i.e., what is the next step)?

*I really hope our group will be able to implement a future research project. The group dynamics are excellent, and we will do an excellent job. It is exciting to think that the results could potentially change how medicine is practiced even if only in one practice at a time.* (Patient Partner 1).

*I would like to see examples of shared decision making in practice, because I have mentioned it to several of my doctors and they acted clueless. I would like to see more options offered from doctors and insurance companies. I don’t know what the next step is.* (Patient Partner 2).

*I believe this group has several goals in mind, all of which are beneficial to the medical field and society. Funding is a major contributor as to how much this group can accomplish at a time but I would like to see this group explore the results of shared decision making regarding the treatment of musculoskeletal pain. I would also like for this group to see what efforts can be made to help fight the opioid epidemic and the research this group needs to make to make that happen.* (Patient Partner 3).

*What are the next steps to consider: project wise and institution wise? The easy answer is to now put together a robust and competitive research plan with attainable worthwhile specific aims. Enough said. Concurrently, I would form a task group to list all the resources that would be nice to have had at the onset. Digital medical records with as much content possible to consider each patient a research project (prospectively and/or retrospectively, pre- and post- intervention patient questionnaires, pain scores throughout treatment, long term follow-up questions, pilot project data. Our group experiences could inform building this medical record database.* (Community Partner)

## Abbreviations

MSK: Musculoskeletal
PCORI®: Patient-Centered Outcomes Research Institute®
GRIPP2: Guidance for Reporting Involvement of Patients and the Public
PPI: Patient and public involvement
P2P: Pipeline to proposal
CER: Comparative effectiveness research
PICOTS: Population, intervention, comparator, outcomes, timeframe, and setting
TAO: Technical Assistance Offices
